# Extracorporeal Photopheresis Stimulates Tissue Repair after Transplantation

**DOI:** 10.1097/TXD.0000000000001812

**Published:** 2025-09-02

**Authors:** Fabiola Arella, Hans J. Schlitt, Paloma Riquelme

**Affiliations:** 1 Laboratory for Transplantation Research, Department of Surgery, University Hospital Regensburg, Regensburg, Germany.

## Abstract

Extracorporeal photopheresis (ECP) is a safe and effective therapy with long-established indications in treating T cell–mediated immune diseases, including steroid refractory graft-versus-host disease and chronic rejection after heart or lung transplantation. The ECP procedure involves collecting autologous peripheral blood leucocytes that are driven into apoptosis before being reinfused intravenously. ECP acts primarily through in situ exposure of recipient dendritic cells and macrophages to apoptotic cells, which then suppress inflammation, promote specific regulatory T-cell responses, and retard fibrosis. Here, we explore the idea that macrophages exposed to apoptotic cell components from photopheresates acquire a tissue-reparative capacity that could be exploited therapeutically. Specifically, we consider innovative applications of ECP in resolving tissue injury after liver transplantation.

## LIVER INJURY AND REPAIR AFTER TRANSPLANTATION

Normal physiological and immunological homeostasis in the liver is abruptly disturbed by donor death or living-donor organ explantation. Deprived of a blood supply, hypoxic injury occurs, and this damage is exacerbated upon reperfusion by oxidative stress.^1^ These combined insults trigger an immediate innate immune response involving tissue-resident macrophages and dendritic cells (DCs) that stimulate a rapid infiltration of the allograft by blood monocytes, neutrophils, and natural killer cells. This early, induced reaction contributes to further tissue injury and recruits T cells, initiating an adaptive immune response. Left untreated, this acute rejection reaction leads to the destruction of transplants within days; however, modern immunosuppression is very effective in controlling early injury, especially the combination of calcineurin inhibitors (CNIs) and high-dose steroids.^2^ Regrettably, many liver transplant recipients with secondary renal failure tolerate CNIs poorly, often resulting in irreversible kidney damage.^3^

When early acute cellular rejection is prevented, transplanted livers enter a regenerative phase characterized by the presence of tissue-reparative macrophages of recipient origin. These cells serve a crucial role in tissue remodeling by removing necrotic debris, stimulating parenchymal regeneration, promoting revascularization, and suppressing T cells within the allograft.^4^ Ideally, tissue repair would lead to restoration of normal immune homeostasis and liver function; however, nonresolving subclinical inflammation is a common outcome.^5^ Hence, we need new therapeutic approaches not only to enhance early tissue regeneration but also to terminate repair processes when injury is resolved. In this article, we examine the role of apoptotic cells in driving tissue regeneration through the differentiation of tissue-reparative macrophages. This leads us to the suggestion that ECP, as an apoptotic cell-based therapy, might have a role in promoting liver repair in the early post-transplant period.

## PRINCIPLES OF EXTRACORPOREAL PHOTOPHERESIS

ECP is an apheretic procedure in which blood leucocytes are collected from a patient before being driven into apoptosis through exposure to a DNA-intercalating agent called 8-methoxypsoralen and UVA irradiation, which causes double-stranded DNA breaks.^6^ Treated leucocytes are immediately reinfused intravenously back into patients, where they presumably die by apoptosis over several days. ECP is a long-established and effective therapy for a variety of T cell–mediated diseases, including graft-versus-host disease.^7^ The precise mechanisms of ECP action are still contested; however, there is an emerging consensus that its primary effect is modifying the behavior of macrophages and DCs.^8^ As discussed, macrophages and DCs play diverse roles in allograft pathology at different phases after transplantation; consequently, the actions of ECP depend on the pathological context in which it is applied.^9-11^

The importance of noninflammatory cell death in maintaining peripheral self-tolerance is well understood. When parenchymal cells become apoptotic, they transfer self-antigen to tissue-resident DCs that migrate in a semi-mature state to lymph nodes. There, DCs interact with potentially autoreactive T cells, leading to their deletion through abortive activation, induction of anergy, or conversion to regulatory T cells.^1,12^ This principle has already been exploited in mouse models, where apoptotic cells delivered by intravenous infusion tolerize recipients to fully mismatched cardiac allografts. This effect has been attributed to splenic red pulp macrophages and CD8α^+^ marginal zone DC that are modified by exposure to apoptotic cell debris.^13^

## APOPTOTIC CELLS INDUCE TISSUE-REPARATIVE MACROPHAGES

Under steady-state conditions, tissue-resident macrophages perform various physiological and immune homeostatic functions, such as the removal of apoptotic cells and the prevention of constitutive inflammation. When they sense a pathogenic insult, tissue-resident macrophages react quickly to eliminate the challenge. Inflammatory cell death, such as necrosis, pyroptosis, or ferroptosis, signifies tissue injury and potently stimulates resident macrophage responses. In contrast, apoptosis is a noninflammatory form of programmed cell death that maintains membrane integrity and suppresses innate reactions through the release of soluble anti-inflammatory factors. Efferocytosis is the phagocytic removal of apoptotic cells by macrophages, which clears dying cells from tissues and induces a tissue-reparative phenotype in macrophages.^14^

Programming of tissue-reparative macrophages by apoptotic cells is now understood in molecular detail, and involves signals delivered by the apoptotic cell microenvironment, by ligands expressed on apoptotic cells, and by the digestion of phagocytic cargo. Apoptotic cells release many soluble factors that contribute to a tolerogenic, proresolving tissue environment. These include cytokines and chemokines (eg, IL-10, transforming growth factor [TGF]-β, C-X3-C motif chemokine ligand 1, IL-38), proresolving mediators (eg, Annexin-A1, thrombospondin-1), and metabolites (eg, spermidine).^15-17^

Apoptotic cells elicit their own engulfment by phagocytes by upregulating ligands for efferocytotic receptors.^18^ Best known among these “eat-me” signals is phosphatidylserine (PS), a phospholipid component of the inner leaflet of cell membranes that translocates the outer leaflet during apoptosis; there, PS is accessible to growth arrest specific 6 and milk fat globule epidermal growth factor 8, which act as intermediaries for TYRO3 protein tyrosine kinase, axl, and mer receptors and α_v_β_5/3_ integrins, respectively. Other apoptotic cell-associated molecules that trigger efferocytosis include calreticulin, phosphatidylethanolamine, high mobility group box 1, sphingosine-1-phosphate, and thrombospondin-1.^19^

Mediators that signal through TAM receptors—namely, MER proto-oncogene, tyrosine kinase, Axl, and TYRO3 protein tyrosine kinase—inhibit the secretion of proinflammatory cytokines and induce lipoxin-4.^20,21^ Other receptors that bind PS include stabilin-2, CD300b, and T-cell membrane protein 4. Scavenger receptors (eg, SR-A family, CD14, CD36) bind a broad range of damage associated molecular patterns and pathogen associated molecular patterns to activate resident macrophages; in addition, activated complement proteins coat apoptotic cell surfaces and are recognized via complement receptors expressed by macrophages, including CR1 (CD35).

Removal of apoptotic cells by macrophages prevents constitutive inflammation in tissues, but their subsequent digestion also generates metabolites that repolarize macrophages toward a noninflammatory, tissue-reparative phenotype. Metabolism of cholesterol from apoptotic cells activates the liver X receptor pathway, further upregulating efferocytic receptors and inhibiting proinflammatory toll-like receptor and nuclear factor kappa-light-chain-enhancer of activated B cells signaling. The metabolism of long-chain fatty acids may activate noncanonical anti-inflammatory pathways involving mitochondrial respiration, NAD^+^, sirtuin-1, and Pbx-1. Fatty acids also stimulate peroxisome proliferator-activated receptors, contributing to macrophage reprogramming and suppression of proinflammatory cytokines.^22,23^ Amino acids liberated by apoptotic cell degradation impact macrophage behavior, especially increased levels of arginine, ornithine, lysine and methionine. Tryptophan metabolism activates metabolic pathways involving indoleamine 2,3-dioxygenase 1 and aryl hydrocarbon receptor, which enhance the induction of proresolving mediators such as IL-10.^24^ Conversion of methionine into S-adenosylmethionine, a substrate for the DNA methyltransferase DNMT3A, links the digestion of apoptotic cells with epigenetic silencing of inflammatory genes. Nucleotides derived from apoptotic cells activate the DNA-dependent protein kinase/mTORC2/phospho-AKT pathway.^15^

The net impact of efferocytosis is reprogramming of macrophages toward a proresolving and prorepair phenotype. Phagocytic uptake of ECP-treated leukocytes by macrophages in inflamed tissue causes STAT6 phosphorylation and the upregulation of CD206, ARG1, and CD301, resulting in an anti-inflammatory phenotype,^25^ which is associated with increased secretion of anti-inflammatory mediators (eg, TGF-β, IL-10, prostaglandin E2) and suppression of inflammatory cytokines (eg, IL-1β, IL-12, TNF-α).^26^

## PROPERTIES OF TISSUE REPAIR MACROPHAGES

Tissue-reparative macrophages typically express CD206, T-cell membrane protein 4, and liver X receptor, as well as anti-inflammatory factors, such as IL-10, TGF-β, and prostaglandin E2. However, there is no definitive description of such macrophages, which are phenotypically and functionally heterogeneous. Indeed, it remains controversial whether there exist distinct subtypes of proresolving macrophages within a single tissue. Key features of proresolving macrophages include high efferocytotic capacity, altered metabolism, and production of so-called proresolving mediators (SPMs).

Proresolving macrophages display a higher capacity for efferocytosis than other macrophages, being able to engulf many apoptotic cells consecutively. Efferocytotic activity enhances macrophages’ capacity for further uptake of apoptotic cells in a feedforward process known as “continual efferocytosis.” Exactly how this mechanism operates is not completely understood; however, the metabolism of ingested apoptotic cell-derived ornithine to putrescine may be crucial.^27^

SPMs are specialized lipid molecules that play a key role in the resolution of inflammation. They prevent the further recruitment of leucocytes to inflamed sites and induce apoptosis in infiltrating immune cells. These mediators derive from polyunsaturated fatty acids, including omega-3 fatty acids such as eicosapentaenoic acid and docosahexaenoic acid, as well as omega-6 fatty acids such as arachidonic acid. SPMs are synthesized through enzymatic pathways involving lipoxygenase, cyclooxygenase, and cytochrome P450 enzymes. SFMs include lipoxins (like LxA4), resolvins (like RvE1 or RvD5), protectins, and maresins.^28^ Notably, some proteins released by macrophages also act as resolution mediators, including Annexin-A1 and developmental endothelial locus-1.^29^

Polarization of macrophages toward a proresolving phenotype is driven by efferocytosis through phagocytic receptors and changes in metabolism related to the degradation of engulfed cells. Consequently, the type of apoptotic cells being phagocytosed, and perhaps the tissue environment where efferocytosis occurs, impacts macrophage phenotype and function.^30^

## EFFECT OF TISSUE-REPARATIVE MACROPHAGES IN LIVER REGENERATION

Liver has a high regenerative capacity owing to the ability of hepatocytes and biliary epithelial cells to proliferate after acute liver injury; however, in chronic liver disease, senescent hepatocytes eventually become exhausted and fibrosis ensues. Liver injury stimulates hepatic stellate cells (HSCs) to secrete extracellular matrix (ECM) components and tissue inhibitors of metalloproteinases that hinder proper tissue remodeling by matrix metalloproteases and exacerbate fibrosis. When liver injury and repair processes fail to resolve, deposition of fibrillary type I and III collagen disrupts normal liver architecture and function, ultimately causing cirrhosis and liver failure.^31^

As the principal resident macrophage population in liver, Kupffer cells are responsible for maintaining tissue homeostasis by clearing dead cells and pathogens arriving via the portal circulation.^32^ When liver is injured, monocytes recruited from blood differentiate into inflammatory macrophages that contribute to parenchymal damage, ECM deposition, and HSC activation. Later, macrophages switch to a proresolving phenotype, partly in response to efferocytosis of apoptotic cells. In particular, liver injury stimulates recruited monocytes and Kupffer cells to become triggering receptor expressed on myeloid cells 2^+^ lipid-associated macrophages that are essential for tissue repair.^33^ Proresolving macrophages control inflammation, remove dead cells, and secrete trophic factors, including platelet-derived growth factor, insulin-like growth factor 1, amphiregulin, and TGF-β. These growth factors support hepatocyte and cholangiocyte proliferation, as well as the remodeling of liver tissue by suppressing HSC activation and reversing excessive ECM deposition.^31^

## THERAPEUTIC MANIPULATION OF TISSUE-REPARATIVE MACROPHAGES IN LIVER INJURY

The central role of tissue repair macrophages in resolving liver injury makes them an attractive therapeutic target. Various approaches are being investigated to increase the density of proresolving macrophages or enhance their function in the damaged liver. One very promising strategy in acute and chronic liver failure is the adoptive transfer of ex vivo–induced tissue-reparative macrophages. In a mouse model of acute paracetamol-induced liver injury, adoptive transfer of IL-4/IL-13-stimulated macrophages reduced inflammation and liver necrosis.^34^ This approach is now being trialed in paracetamol-induced acute liver injury.^35^ In the chronic setting, autologous macrophage therapy for liver cirrhosis has also been investigated in preclinical models^36^ and clinical trials.^37^

**FIGURE 1.**
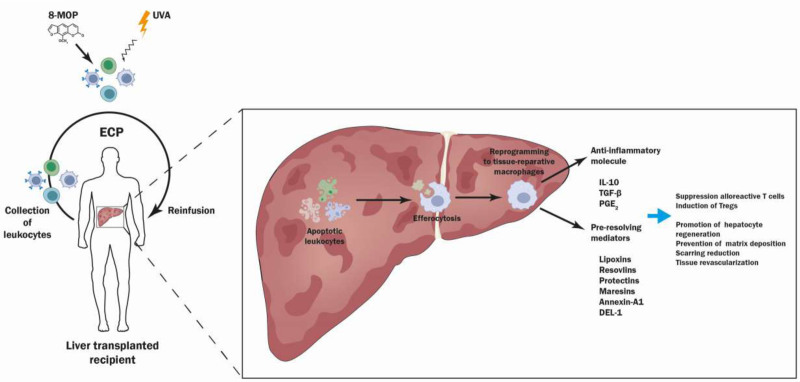
ECP-induced reprogramming of macrophages after liver transplantation. We hypothesize that clearance of apoptotic remnants of leucocytes from photopheresates drives the development of tissue-reparative macrophages within transplanted livers. These reprogrammed macrophages may then contribute to a local immunosuppressive microenvironment and tissue remodeling. DEL-1, developmental endothelial locus-1; ECP, extracorporeal photopheresis; 8-MOP, 8-methoxypsoralen; IL, interleukin; PGE-2, prostaglandin E2; TGF-β, transforming growth factor-β; Treg, regulatory T cell.

An alternative therapeutic approach involves nanoparticle-encapsulated drugs to modify macrophages in vivo. Using liposomes to deliver dexamethasone to macrophages mitigated disease in mice with acute or chronic liver injury.^38^ Similar strategies include encapsulated PS or apoptotic vesicles.^39^ SuperMApo is a mixture of factors secreted by proresolving macrophages after coculture with apoptotic cells that is active in mouse models of inflammation.^40^ Given the potential therapeutic benefit of supporting proresolving macrophages after acute liver injury, and knowing that apoptotic cells drive the physiological development of proresolving macrophages, we propose that ECP could be valuable in early postoperative management of liver transplant recipients (Figure 1).

## EXTRACORPOREAL PHOTOPHERESIS AFTER LIVER TRANSPLANTATION

Chronic renal impairment after liver transplantation is a common complication that significantly impacts morbidity and mortality. Around two-thirds of patients undergoing liver transplantation at our center have severe hepatorenal dysfunction, making them more vulnerable to the nephrotoxic effects of CNIs. Clinical protocols aimed at reducing early CNI exposure preserve and restore the filtrative capacity of kidneys in liver transplant recipients; however, this is achieved at the cost of a higher rate of acute cellular rejection. Using ECP as an induction therapy after early liver transplantation offers an attractive solution to the concurrent challenges of CNI nephrotoxicity, risk of rejection, and the need for tissue repair.

To avoid CNI nephrotoxicity in liver transplant recipients, our center introduced a “bottom-up” strategy that delays the introduction of tacrolimus until 8–10 d posttransplant. The PATRON07 Study showed that early CNI avoidance significantly reduced the incidence of chronic renal failure in liver transplant recipients.^41^ In this single-arm study, 27 patients with preoperative renal failure (estimated glomerular filtration rate 24 ± 17.1 mL/min) and high laboratory Model for End-stage Liver Disease scores received bottom-up immunosuppression. The 1y overall survival was 93% and the mean estimated glomerular filtration rate increased significantly from baseline to 1 y. The major drawback of this protocol was the relatively high rate (37%) of biopsy-proven acute rejections.

ECP has been used in other contexts to support temporary minimization or withdrawal of immunosuppression in transplant recipients while treating infections or posttransplant lymphoproliferative disorder^42^ although studies in liver transplantation are sparse.^43^ Additionally, ECP induction showed promise as a CNI-avoidance strategy in cardiac transplant recipients at high risk of infection or recurrent malignancy.^44^ Urbani et al investigated ECP as a CNI-sparing therapy after liver transplantation. Although they found no differences in acute rejection rates, CNI could be introduced later in the ECP-treated group, which showed better 6- and 12-mo survival.^45^ We propose that this concept could be extended to managing liver transplant recipients with preexisting renal failure by avoiding early CNI exposure while also preventing acute rejection and supporting tissue repair processes.

**FIGURE 2.**
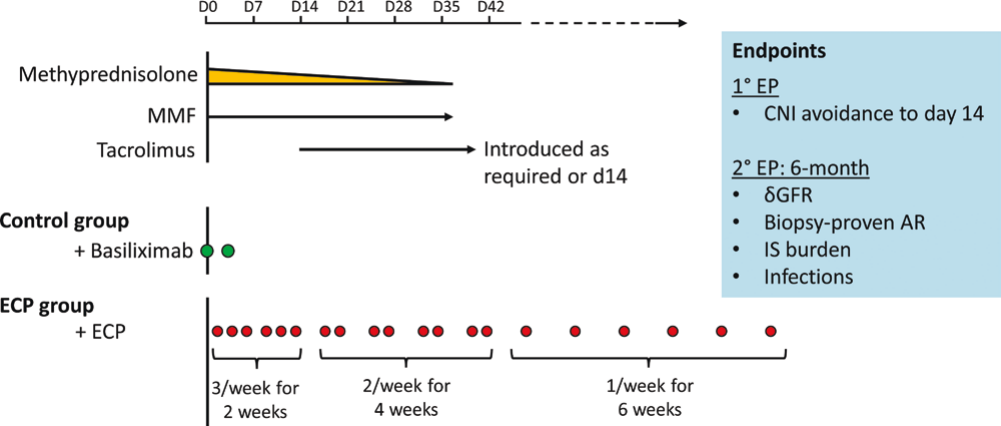
A proposed clinical trial design for using ECP as a CNI-sparing therapy in liver transplant patients with renal failure. Liver transplant patients will be treated with an intensive ECP therapy regimen for the first 6 wk, reducing to once weekly for a further 6 wk. Patients in the ECP-treated and control arms will receive standard-of-care immunosuppression, except that IL-2R blockade (basiliximab) will be omitted in the ECP group. The primary endpoint will be time-to-introduction of tacrolimus. AR, acute rejection; CNI, calcineurin inhibitor; ECP, extracorporeal photopheresis; GFR, glomerular filtration rate; IL, interleukin; IS, immunosuppression; MMF, mycophenolate mofetil.

To this end, our center is currently designing a randomized controlled trial in which adult liver transplant recipients with preexisting renal impairment will be treated with standard-dose methylprednisolone and mycophenolate mofetil plus high-intensity ECP treatment for up to 6 wk. Standard treatment with tacrolimus will only be introduced on day 14, unless signs of rejection indicate otherwise. Basiliximab induction, which is standard-of-care at our center, will be omitted in the ECP treatment arm because IL-2R blockade interferes with T-cell regulation. Control group patients will receive standard triple immunosuppression plus Basiliximab induction. The primary endpoint of this study will be the proportion of patients spared from CNIs for 14 d. Renal function at 6 mo posttransplant and absence of subclinical inflammation in protocol biopsies will be important secondary endpoints (Figure 2).

## CONTRIBUTION OF THE exTra CONSORTIUM

In conclusion, ECP is a safe and effective therapy with established indications in heart or lung transplantation. Through a deeper understanding of its immunological actions, the exTra consortium aims to extend indications for ECP in solid organ transplantation.^46-48^ As part of exTra, we are working with groups across Europe to harmonize methods for studying the effects of ECP in vitro and in vivo.^49,50^ Through the collaborative efforts of exTra, we hope to assemble a panel of reliable assays to guide the safe introduction of ECP induction therapy in liver transplantation.

## References

[1] Adler AJ Marsh DW Yochum GS. CD4+ T cell tolerance to parenchymal self-antigens requires presentation by bone marrow-derived antigen-presenting cells. J Exp Med. 1998;187:1555–1564. 9584134 10.1084/jem.187.10.1555 PMC2212299

[2] Hutchinson JA Geissler EK. Now or never? The case for cell-based immunosuppression in kidney transplantation. Kidney Int. 2015;87:1116–1124. 25738251 10.1038/ki.2015.50

[3] Schnitzbauer AA Doenecke A Sothmann JL. Improved outcome after ‘bottom-up’ immunosuppression in liver transplant recipients with preoperative renal impairment. Eur Surg Res. 2010;45:356–367. 21088426 10.1159/000321702

[4] Wynn TA Vannella KM. Macrophages in tissue repair, regeneration, and fibrosis. Immunity. 2016;44:450–462. 26982353 10.1016/j.immuni.2016.02.015 PMC4794754

[5] Vionnet J Miquel R Abraldes JG. Non-invasive alloimmune risk stratification of long-term liver transplant recipients. J Hepatol. 2021;75:1409–1419. 34437910 10.1016/j.jhep.2021.08.007

[6] Stępień J Eggenhofer E. ECP-induced apoptosis: how noninflammatory cell death counterbalances ischemia-reperfusion injury. Transplant Direct. 2025;11:e1816. 10.1097/TXD.0000000000001816 PMC12410312 40919453

[7] Ahrens N Geissler EK Witt V. European reflections on new indications for extracorporeal photopheresis in solid organ transplantation. Transplantation. 2018;102:1279–1283. 29664769 10.1097/TP.0000000000002244

[8] Hutchinson JA Benazzo A. Extracorporeal photopheresis suppresses transplant fibrosis by inducing decorin expression in alveolar macrophages. Transplantation. 2023;107:1010–1012. 37097979 10.1097/TP.0000000000004536

[9] Nogueira F van Ham SM ten Brinke A. Extracorporeal photopheresis in solid organ transplantation: modulating B cell responses to improve graft survival. Transplant Direct. 2025;11:e1833. 10.1097/TXD.0000000000001833 PMC13155511 42111684

[10] Tocco C Ochando J. Potential Impact of Extracorporeal Photopheresis on Trained Immunity and Organ Transplant Acceptance. Transplant Direct. 2025;11:e1835. 10.1097/TXD.0000000000001835 PMC12410317 40919456

[11] Garcia-Almeida JH Heger L Hackstein H. Extracorporeal photopheresis: secreted factors that promote immunomodulation. Transplant Direct. 2025;11:e1840. 10.1097/TXD.0000000000001840 PMC13221125 42222516

[12] Kurts C Heath WR Kosaka H. The peripheral deletion of autoreactive CD8+ T cells induced by cross-presentation of self-antigens involves signaling through CD95 (Fas, Apo-1). J Exp Med. 1998;188:415–420. 9670055 10.1084/jem.188.2.415 PMC2212451

[13] Wang Z Larregina AT Shufesky WJ. Use of the inhibitory effect of apoptotic cells on dendritic cells for graft survival via T-cell deletion and regulatory T cells. Am J Transplant. 2006;6:1297–1311. 16686754 10.1111/j.1600-6143.2006.01308.x

[14] Roberts AW Lee BL Deguine J. Tissue-resident macrophages are locally programmed for silent clearance of apoptotic cells. Immunity. 2017;47:913–927.e6. 29150239 10.1016/j.immuni.2017.10.006 PMC5728676

[15] Saas P Vetter M Maraux M. Resolution therapy: harnessing efferocytic macrophages to trigger the resolution of inflammation. Front Immunol. 2022;13:1021413. 36389733 10.3389/fimmu.2022.1021413 PMC9651061

[16] Elliott MR Koster KM Murphy PS. Efferocytosis signaling in the regulation of macrophage inflammatory responses. J Immunol. 2017;198:1387–1394. 28167649 10.4049/jimmunol.1601520 PMC5301545

[17] Mora J Schlemmer A Wittig I. Interleukin-38 is released from apoptotic cells to limit inflammatory macrophage responses. J Mol Cell Biol. 2016;8:426–438. 26892022 10.1093/jmcb/mjw006

[18] Doran AC Yurdagul A Jr Tabas I. Efferocytosis in health and disease. Nat Rev Immunol. 2020;20:254–267. 31822793 10.1038/s41577-019-0240-6 PMC7667664

[19] Cockram TOJ Dundee JM Popescu AS. The phagocytic code regulating phagocytosis of mammalian cells. Front Immunol. 2021;12:629979. 34177884 10.3389/fimmu.2021.629979 PMC8220072

[20] Triantafyllou E Pop OT Possamai LA. MerTK expressing hepatic macrophages promote the resolution of inflammation in acute liver failure. Gut. 2018;67:333–347. 28450389 10.1136/gutjnl-2016-313615 PMC5868289

[21] Cai B Kasikara C Doran AC. MerTK signaling in macrophages promotes the synthesis of inflammation resolution mediators by suppressing CaMKII activity. Sci Signal. 2018;11:eaar3721. 30254055 10.1126/scisignal.aar3721 PMC6171110

[22] A-Gonzalez N Bensinger SJ Hong C. Apoptotic cells promote their own clearance and immune tolerance through activation of the nuclear receptor LXR. Immunity. 2009;31:245–258. 19646905 10.1016/j.immuni.2009.06.018 PMC2791787

[23] Zhang S Weinberg S DeBerge M. Efferocytosis fuels requirements of fatty acid oxidation and the electron transport chain to polarize macrophages for tissue repair. Cell Metab. 2019;29:443–456.e5. 30595481 10.1016/j.cmet.2018.12.004 PMC6471613

[24] Sukka SR Ampomah PB Darville LNF. Efferocytosis drives a tryptophan metabolism pathway in macrophages to promote tissue resolution. Nat Metab. 2024;6:1736–1755. 39242914 10.1038/s42255-024-01115-7 PMC11734744

[25] Braun LM Giesler S Andrieux G. Adiponectin reduces immune checkpoint inhibitor-induced inflammation without blocking anti-tumor immunity. Cancer Cell. 2025;43:269–291.e19. 39933899 10.1016/j.ccell.2025.01.004

[26] Fadok VA Bratton DL Konowal A. Macrophages that have ingested apoptotic cells in vitro inhibit proinflammatory cytokine production through autocrine/paracrine mechanisms involving TGF-beta, PGE2, and PAF. J Clin Invest. 1998;101:890–898. 9466984 10.1172/JCI1112 PMC508637

[27] Yurdagul A Jr Subramanian M Wang X. Macrophage metabolism of apoptotic cell-derived arginine promotes continual efferocytosis and resolution of injury. Cell Metab. 2020;31:518–533.e10. 32004476 10.1016/j.cmet.2020.01.001 PMC7173557

[28] Julliard WA Myo YPA Perelas A. Specialized pro-resolving mediators as modulators of immune responses. Semin Immunol. 2022;59:101605. 35660338 10.1016/j.smim.2022.101605 PMC9962762

[29] Fredman G. DELineating resolution of inflammation. Nat Immunol. 2019;20:2–3. 30538337 10.1038/s41590-018-0278-9

[30] Liebold I Al Jawazneh A Casar C. Apoptotic cell identity induces distinct functional responses to IL-4 in efferocytic macrophages. Science. 2024;384:eabo7027. 38574142 10.1126/science.abo7027

[31] Campana L Esser H Huch M. Liver regeneration and inflammation: from fundamental science to clinical applications. Nat Rev Mol Cell Biol. 2021;22:608–624. 34079104 10.1038/s41580-021-00373-7

[32] Guilliams M Bonnardel J Haest B. Spatial proteogenomics reveals distinct and evolutionarily conserved hepatic macrophage niches. Cell. 2022;185:379–396.e38. 35021063 10.1016/j.cell.2021.12.018 PMC8809252

[33] De Ponti FF Bujko A Liu Z. Spatially restricted and ontogenically distinct hepatic macrophages are required for tissue repair. Immunity. 2025;58:362–380.e10. 39862865 10.1016/j.immuni.2025.01.002

[34] Starkey Lewis P Campana L Aleksieva N. Alternatively activated macrophages promote resolution of necrosis following acute liver injury. J Hepatol. 2020;73:349–360. 32169610 10.1016/j.jhep.2020.02.031 PMC7378576

[35] Humphries C Addison M Aithal G. Macrophage Therapy for Acute Liver Injury (MAIL): a study protocol for a phase 1 randomised, open-label, dose-escalation study to evaluate safety, tolerability and activity of allogeneic alternatively activated macrophages in patients with paracetamol-induced acute liver injury in the UK. BMJ Open. 2024;14:e089417. 10.1136/bmjopen-2024-089417 PMC11628987 39653576

[36] Thomas JA Pope C Wojtacha D. Macrophage therapy for murine liver fibrosis recruits host effector cells improving fibrosis, regeneration, and function. Hepatology. 2011;53:2003–2015. 21433043 10.1002/hep.24315

[37] Brennan PN MacMillan M Manship T. Autologous macrophage therapy for liver cirrhosis: a phase 2 open-label randomized controlled trial. Nat Med. 2025;31:979–987. 39794616 10.1038/s41591-024-03406-8 PMC11922741

[38] Bartneck M Scheyda KM Warzecha KT. Fluorescent cell-traceable dexamethasone-loaded liposomes for the treatment of inflammatory liver diseases. Biomaterials. 2015;37:367–382. 25453965 10.1016/j.biomaterials.2014.10.030

[39] Zhong Z Cui XL Tan KJ. Apoptotic vesicles (apoVs) derived from fibroblast-converted hepatocyte-like cells effectively ameliorate liver fibrosis. J Nanobiotechnol. 2024;22:541. 10.1186/s12951-024-02824-7 PMC11375929 39238002

[40] Bonnefoy F Gauthier T Vallion R. Factors produced by macrophages eliminating apoptotic cells demonstrate pro-resolutive properties and terminate ongoing inflammation. Front Immunol. 2018;9:2586. 30542342 10.3389/fimmu.2018.02586 PMC6277856

[41] Schnitzbauer AA Sothmann J Baier L. Calcineurin inhibitor free de novo immunosuppression in liver transplant recipients with pretransplant renal impairment: results of a pilot study (PATRON07). Transplantation. 2015;99:2565–2575. 26018348 10.1097/TP.0000000000000779

[42] Barten MJ Fisher AJ Hertig A. The use of extracorporeal photopheresis in solid organ transplantation-current status and future directions. Am J Transplant. 2024;24:1731–1741. 38490642 10.1016/j.ajt.2024.03.012

[43] Urbani L Mazzoni A Colombatto P. Potential applications of extracorporeal photopheresis in liver transplantation. Transplant Proc. 2008;40:1175–1178. 18555142 10.1016/j.transproceed.2008.03.071

[44] Gökler J Aliabadi-Zuckermann A Zuckermann A. Extracorporeal photopheresis with low-dose immunosuppression in high-risk heart transplant patients-a pilot study. Transpl Int. 2022;35:10320. 35401042 10.3389/ti.2022.10320 PMC8983826

[45] Urbani L Mazzoni A De Simone P. Avoiding calcineurin inhibitors in the early post-operative course in high-risk liver transplant recipients: the role of extracorporeal photopheresis. J Clin Apher. 2007;22:187–194. 17294458 10.1002/jca.20111

[46] Parsonidis P Wekerle T. Extracorporeal photopheresis: does it have a potential place among cell-based therapies? Transplant Direct. 2025;11:e1808. 10.1097/TXD.0000000000001808 PMC12410314 40919458

[47] Nicoli M Rovira J Diekmann F. Exploring the role of extracorporeal photopheresis in kidney transplant management. Transplant Direct. 2025;11:e1809. 10.1097/TXD.0000000000001809 PMC12410308 40919452

[48] Alemanno S Jaksch P Benazzo A. Extracorporeal photopheresis in lung transplantation: present applications and emerging research. Transplant Direct. 2025;11:e1831. 10.1097/TXD.0000000000001831 PMC12410313 40919454

[49] Morgado I Vinnakota JM Zeiser R. Extracorporeal photopheresis: from animal models to clinical practice. Transplant Direct. 2025;11:e1824. 10.1097/TXD.0000000000001824 PMC13155520 42111683

[50] Veltman H Martinez-Caceres E Iglesias-Escudero M. Measuring the immunomodulatory effects of extracorporeal photopheresis in solid organ transplantation. Transplant Direct. 2025;11:e1817. 10.1097/TXD.0000000000001817 PMC12410318 40919455

